# Downregulation of SIRT1 signaling underlies hepatic autophagy impairment in glycogen storage disease type Ia

**DOI:** 10.1371/journal.pgen.1006819

**Published:** 2017-05-30

**Authors:** Jun-Ho Cho, Goo-Young Kim, Chi-Jiunn Pan, Javier Anduaga, Eui-Ju Choi, Brian C. Mansfield, Janice Y. Chou

**Affiliations:** 1 Section on Cellular Differentiation, Eunice Kennedy Shriver National Institute of Child Health and Human Development, National Institutes of Health, Bethesda, Maryland, United States of America; 2 Laboratory of Cell Death and Human Diseases, Department of Life Sciences, Korea University, Seoul, South Korea; 3 Foundation Fighting Blindness, Columbia, Maryland, United States of America; University College London, UNITED KINGDOM

## Abstract

A deficiency in glucose-6-phosphatase-α (G6Pase-α) in glycogen storage disease type Ia (GSD-Ia) leads to impaired glucose homeostasis and metabolic manifestations including hepatomegaly caused by increased glycogen and neutral fat accumulation. A recent report showed that G6Pase-α deficiency causes impairment in autophagy, a recycling process important for cellular metabolism. However, the molecular mechanism underlying defective autophagy is unclear. Here we show that in mice, liver-specific knockout of G6Pase-α (L-*G6pc*-/-) leads to downregulation of sirtuin 1 (SIRT1) signaling that activates autophagy via deacetylation of autophagy-related (ATG) proteins and forkhead box O (FoxO) family of transcriptional factors which transactivate autophagy genes. Consistently, defective autophagy in G6Pase-α-deficient liver is characterized by attenuated expressions of autophagy components, increased acetylation of ATG5 and ATG7, decreased conjugation of ATG5 and ATG12, and reduced autophagic flux. We further show that hepatic G6Pase-α deficiency results in activation of carbohydrate response element-binding protein, a lipogenic transcription factor, increased expression of peroxisome proliferator-activated receptor-γ (PPAR-γ), a lipid regulator, and suppressed expression of PPAR-α, a master regulator of fatty acid β-oxidation, all contributing to hepatic steatosis and downregulation of SIRT1 expression. An adenovirus vector-mediated increase in hepatic SIRT1 expression corrects autophagy defects but does not rectify metabolic abnormalities associated with G6Pase-α deficiency. Importantly, a recombinant adeno-associated virus (rAAV) vector-mediated restoration of hepatic G6Pase-α expression corrects metabolic abnormalities, restores SIRT1-FoxO signaling, and normalizes defective autophagy. Taken together, these data show that hepatic G6Pase-α deficiency-mediated down-regulation of SIRT1 signaling underlies defective hepatic autophagy in GSD-Ia.

## Author summary

GSD-Ia is an autosomal recessive metabolic disorder caused by a deficiency in G6Pase-α, a key enzyme in maintaining blood glucose levels between meals. Despite strong compliance to dietary therapies, GSD-Ia patients continue manifesting metabolic aberrations including excessive accumulation of glycogen and lipid in the liver. Recently, G6Pase-α deficiency has been linked to impairment in autophagy, a recycling process essential for cellular homeostasis. However, the underlying mechanism is unclear. In this study, we show that hepatic G6Pase-α deficiency alters the activity and/or expression of several lipid regulators, leading to hepatic steatosis and reduced expression of SIRT1, an enzyme that regulates the activity of many proteins via deacetylation. The impaired SIRT1 signaling increases the acetylation of ATG proteins critical for autophagic vesicle elongation, and reduces the activity of FoxO factors that can induce autophagy genes. Consistently, the G6Pase-α-deficient liver exhibits autophagy impairment characterized by attenuated expression of many autophagy components, defective autophagic vesicle elongation, impaired autophagosome formation, and reduced autophagy flux. Importantly, SIRT1 overexpression in G6Pase-α-deficient liver corrects autophagy deficiency. Finally, restoration of hepatic G6Pase-α expression corrects metabolic abnormalities, restores SIRT1-FoxO signaling, and normalizes defective autophagy. Collectively, hepatic G6Pase-α deficiency-mediated down-regulation of SIRT1 signaling underlies defective hepatic autophagy in GSD-Ia.

## Introduction

Glycogen storage disease type Ia (GSD-Ia, MIM232200) is caused by a deficiency in glucose-6-phosphatase-α (G6Pase-α or G6PC), an enzyme expressed primarily in liver, kidney, and intestine [1]. G6Pase-α catalyzes the hydrolysis of glucose-6-phosphate (G6P) to glucose and phosphate in the terminal step of glycogenolysis and gluconeogenesis and is a key enzyme for endogenous production of blood glucose [1]. GSD-Ia patients manifest impaired glucose homeostasis characterized by fasting hypoglycemia, hepatomegaly, hypercholesterolemia, hypertriglyceridemia, hyperuricemia, lactic acidemia, and growth retardation [1]. Hepatomegaly is caused by excessive glycogen and neutral fat accumulation [1]. Strict compliance with dietary therapies has enabled GSD-Ia patients to attain near normal growth and pubertal development [1]. However, long-term complications, including hepatocellular adenoma (HCA) still occur in metabolically compensated GSD-Ia patients [1]. We have previously generated a global *G6pc*-/- mouse line that mimics the phenotype of human GSD-Ia. However, even under intensive glucose therapy, the *G6pc*-/- mice rarely survive to weaning, making the follow-up study of metabolic aberrations difficult [2]. On the other hand, the liver-specific *G6pc*-knockout (L-*G6pc*-/-) mice survive to adulthood and develop HCA [3], offering a suitable model to study the long-term manifestations of hepatic G6Pase-α deficiency.

Macroautophagy (or autophagy) is a recycling mechanism that produces energy and building blocks through lysosomal degradation of intracellular proteins and organelles in times of nutrient deprivation and environmental stresses [4]. Autophagy is involved in the breakdown of lipid droplets via a selective form of autophagy called lipophagy [5]. Autophagy also functions as a cellular quality-control system by eliminating protein aggregates and defective organelles [6]. Since the liver plays essential roles in energy homeostasis, hepatic autophagy deficiency has been linked to many metabolic disorders including diabetes, obesity, non-alcoholic fatty liver disease and hepatocarcinogenesis [5].

Several energy sensing pathways, including mammalian target of rapamycin (mTOR), AMP-activated protein kinase (AMPK), and sirtuin 1 (SIRT1) regulate autophagy pathway [7]. Autophagy occurs stepwise from initiation, vesicle nucleation, vesicle elongation, and to final fusion of the autophagosome with a lysosome for component degradation [8]. Autophagy initiation is mediated by signaling via the Unc-51-like kinase 1 (ULK1) complex [8]. Under nutrient-rich conditions, mTOR plays a central role as a negative regulator of autophagy via inhibitory phosphorylation of ULK1 [9]. In contrast, AMPK, an energy sensor promotes autophagy by activating phosphorylation of ULK1 or inhibiting the mTOR pathway [7]. Autophagy can also be regulated by SIRT1, a deacetylase that is activated by increased expression as well as by increased cellular NAD^+^ levels in response to nutrient starvation [7]. Studies have shown that SIRT1 regulates autophagy directly via deacetylation of autophagy-related (ATG) proteins and indirectly via deacetylation and activation of forkhead box O (FoxO) members which transactivate autophagy genes [10].

Using *G6pc*-deficient cell lines and young *G6pc*-/- mice, Farah et al. [11] have recently shown that G6Pase-α deficiency leads to autophagy impairment and suggested that mTOR signaling may play a role in autophagy deficiency seen in GSD-Ia. However, the mechanism underlying autophagy deficiency in GSD-Ia remains unclear. Using adult L-*G6pc*-/- mice, we now show that the G6Pase-α-deficient liver displays impaired autophagy characterized by attenuated expression of autophagy components, impaired autophagosome formation, and reduced autophagy flux. We further show that the expression of SIRT1 and FoxO3a is reduced in G6Pase-α-deficient livers. Interestingly, we show that hepatic G6Pase-α deficiency leads to activation of carbohydrate response element-binding protein (ChREBP) signaling, increase in peroxisome proliferator-activated receptor-γ (PPAR-γ) expression, and suppression in PPAR-α expression that all contribute to hepatic steatosis and downregulation of SIRT1 expression. Importantly, hepatic SIRT1 overexpression restores the expression of autophagy components and normalizes autophagic flux in G6Pase-α-deficient livers, while inhibition of mTOR signaling by rapamycin fails to correct defective hepatic autophagy. Thus, our results indicate that downregulation of SIRT1 signaling underlie autophagy deficiency in GSD-Ia. Finally, we show that restoration of hepatic G6Pase-α expression corrects metabolic abnormalities, restores SIRT1-FoxO signaling and normalizes defective autophagy.

## Results

### Impaired hepatic autophagy pathway in L-*G6pc*-/- mice

**Fig 1:**
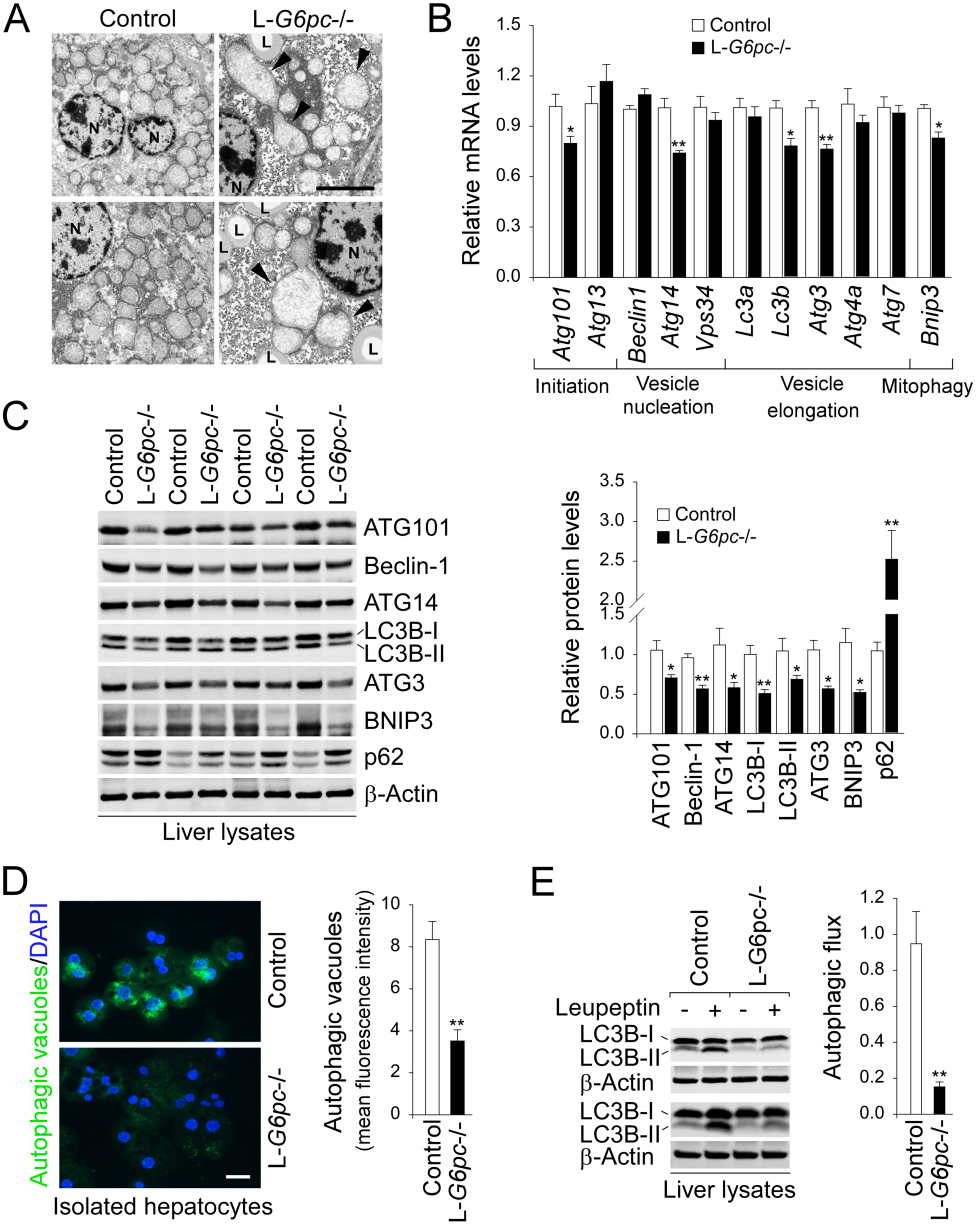
Impaired hepatic autophagy in L-*G6pc-/-* mice. (A) Electron micrographs of hepatocytes in the livers of control and L-*G6pc*-/- mice. Arrowheads indicate deformed mitochondria. N: nucleus, L: lipid droplet. Scale bar, 5 μm. (B) Quantification of mRNA for hepatic autophagy components by real-time RT-PCR (n = 8). (C) Western blots of hepatic ATG101, Beclin-1, ATG14, LC3B, ATG3, BNIP3, p62 and β-actin, and densitometry analysis (n = 8). (D) Immunofluorescence analysis of autophagic vacuoles (green) and DAPI-stained nuclei (blue) in hepatocytes isolated from control and L-*G6pc*-/- mice after 24 hours of fast, and quantification of autophagy vacuoles by flow cytometry (n = 4). Scale bar, 25 μm. (E) Western blots for hepatic LC3B-II and β-actin in mice treated with either saline (-) or leupeptin (+) and determination of autophagic flux (n = 4). Data represent the mean ± SEM. *P < 0.05, **P < 0.005.

The young global *G6pc-*/- mice display signs of hepatic autophagy deficiency [11] but the mice die young, making studies of long-term consequences of autophagy deficiency difficult. To delineate the mechanism underlying autophagy deficiency in GSD-Ia, we generated L-*G6pc*-/- mice which survived to adulthood as previously described [3]. The mice were genotyped (S1A Fig) and liver-specific deletion of the *G6pc* gene was confirmed by Western blot analysis (S1B Fig). Studies have shown that autophagy-deficient livers frequently harbor morphologically abnormal and defective mitochondria [12, 13]. Consistently, we detected many swollen and deformed mitochondria in the livers of L-*G6pc*-/- mice (Fig 1A), suggesting impaired autophagy.

Autophagy occurs stepwise from initiation, vesicle nucleation, vesicle elongation, to fusion of the autophagosome-lysosome for component degradation [8]. In the livers of L-*G6pc*-/- mice, impaired autophagy was evidenced by decreased expression of several components of the autophagy pathway, including initiation (ATG101), vesicle nucleation (ATG14), elongation (LC3B or microtubule-associated protein 1 light chain 3B, and ATG3), and mitophagy (BNIP3 or BCL2/adenovirus E1B 19 kDa protein-interacting protein 3) (Fig 1B and 1C). A key step in vesicle nucleation of autophagy is complex formation of Beclin-1 with Vps34 (class III phosphatidylinositol 3-kinase) and ATG14. Interestingly, the decrease in Beclin-1 protein in the livers of L-*G6pc*-/- mice (Fig 1C) was not accompanied with a corresponding decrease in *Beclin1* transcripts (Fig 1B). Studies have shown that reduced expression of complex components can de-stabilize Beclin-1 [14], raising the possibility that reduced expression of ATG14 might lead to increased Beclin-1 protein turnover in the livers of L-*G6pc*-/- mice. During vesicle elongation of autophagy, the non-lipidated form of LC3-I is converted to phosphatidylethanolamine-conjugated LC3-II, a marker of autophagosome formation [8]. Compared to control livers, both levels of LC3B isoform, LC3B-I and LC3B-II, were reduced in the livers of L-*G6pc*-/- mice (Fig 1C), consistent with impaired autophagosome formation. We further showed that levels of p62, a selective substrate for autophagy [4] were markedly increased in the livers of L-*G6pc*-/- mice compared to the controls (Fig 1C). Furthermore, compared to the controls, the hepatocytes isolated from the livers of L-*G6pc*-/- mice harbored reduced numbers of autophagic vacuoles as shown by reduced staining for cyto-ID, a specific dye for autophagosomes and autophagolysosomes [15] (Fig 1D). To confirm that our observations for impaired hepatic autophagy in L-*G6pc*-/- mice resulted from reduced autophagosome formation but not from increased lysosomal clearance of autophagosome, we examined autophagic flux *in-vivo* by examining hepatic LC3B-II levels in control and L-*G6pc*-/- mice treated with either saline or leupeptin, a lysosomal inhibitor. Our results showed that autophagic flux was significantly attenuated in the livers of L-*G6pc*-/- mice, compared to control livers (Fig 1E).

### Impaired hepatic SIRT1-FoxO signaling in L-*G6pc*-/- mice

**Fig 2:**
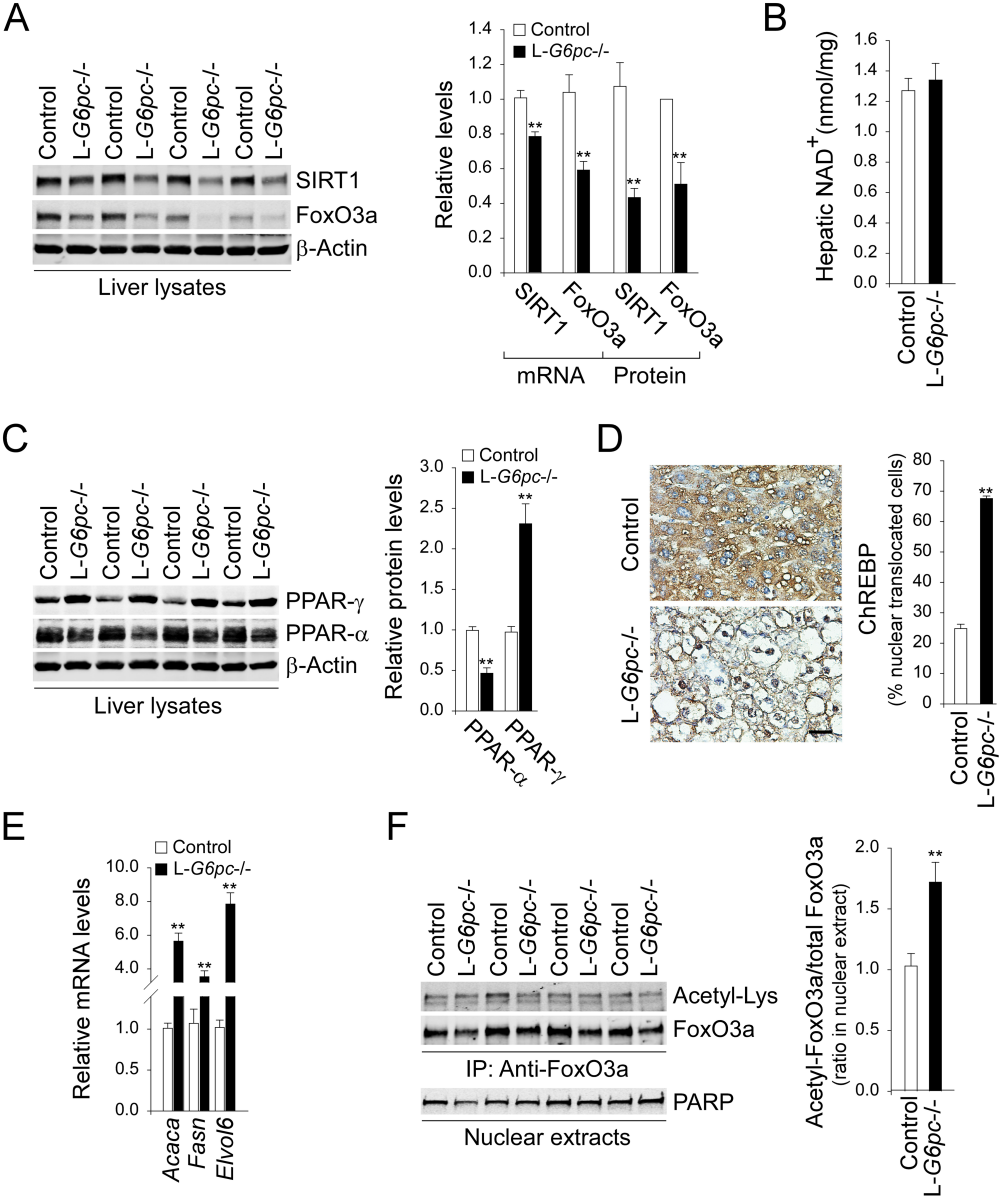
Impaired hepatic SIRT1-FoxO signaling in L-*G6pc*-/- mice. (A) Western blots and densitometry analysis (n = 5), and quantification of mRNA for hepatic SIRT1 and FoxO3a (n = 8). (B) Hepatic NAD^+^ levels (n = 9). (C) Western blots and densitometry analysis of PPAR-γ, PPAR-α and β-actin (n = 5). (D) Immunohistochemical analysis of hepatic ChREBP and quantification of nuclear ChREBP-translocated cells (n = 4). Scale bar, 25 μm. (E) Quantification of mRNA for hepatic *Acaca*, *Fasn and Elovl6* by real-time RT-PCR (n = 6). (F) Western blots of acetylated and total FoxO3a after immunoprecipitation of nuclear extracts using anti-FoxO3a, and quantification of the acetylated FoxO3a/total FoxO3a (n = 5). Data represent the mean ± SEM. *P < 0.05, **P < 0.005.

The impaired autophagy in the livers of L-*G6pc*-/- mice was characterized by decreased expression of many autophagy components (Fig 1B and 1C). Since the FoxO factors stimulate the transcription of many *Atg* genes, and SIRT1 can deacetylase and activate the transcriptional activity of FoxO factors [8, 10], we examined the SIRT1-FoxO signaling pathway. In the livers of L-*G6pc*-/- mice, levels of mRNA and protein of SIRT1 and FoxO3a were decreased, compared to controls (Fig 2A). The transcription of SIRT1, a NAD^+^-dependent deacetylase can be stimulated by PPAR-α, and down-regulated by PPAR-γ and ChREBP [16]. Hepatic NAD^+^ levels, an essential cofactor of SIRT1 were similar between control and L-*G6pc*-/- mice (Fig 2B). However, compared to controls, levels of PPAR-γ were higher in the livers of L-*G6pc*-/- mice whereas those of PPAR-α were lower (Fig 2C). Moreover, the livers of L-*G6pc*-/- mice exhibited activated ChREBP signaling, demonstrated by increased nuclear-translocated ChREBP protein (Fig 2D and S2 Fig) and increased expression of ChREBP target lipogenic genes including acetyl-Co A carboxylase-α (*Acaca*), fatty acid synthase (*Fasn*), and elongation of very long chain fatty acids protein 6 (*Elovl6*) [17] (Fig 2E). These results are in good agreement with the reduced expression of SIRT1 in the livers of L-*G6pc*-/- mice. Taken together, these results suggest that the changes of factors that lead to hepatic steatosis contribute to down-regulation of hepatic SIRT1 in L-*G6pc*-/- mice. Consistent with the attenuated expression of SIRT1, the ratios of acetylated FoxO3a (inactive form) to total FoxO3a in nuclear extracts of the livers of L-*G6pc*-/- mice were higher than those of control livers (Fig 2F), demonstrating that the relative levels of active FoxO3a were reduced in the livers of L-*G6pc*-/- mice.

### Increased hepatic acetylated ATG proteins in L-*G6pc*-/- mice

**Fig 3:**
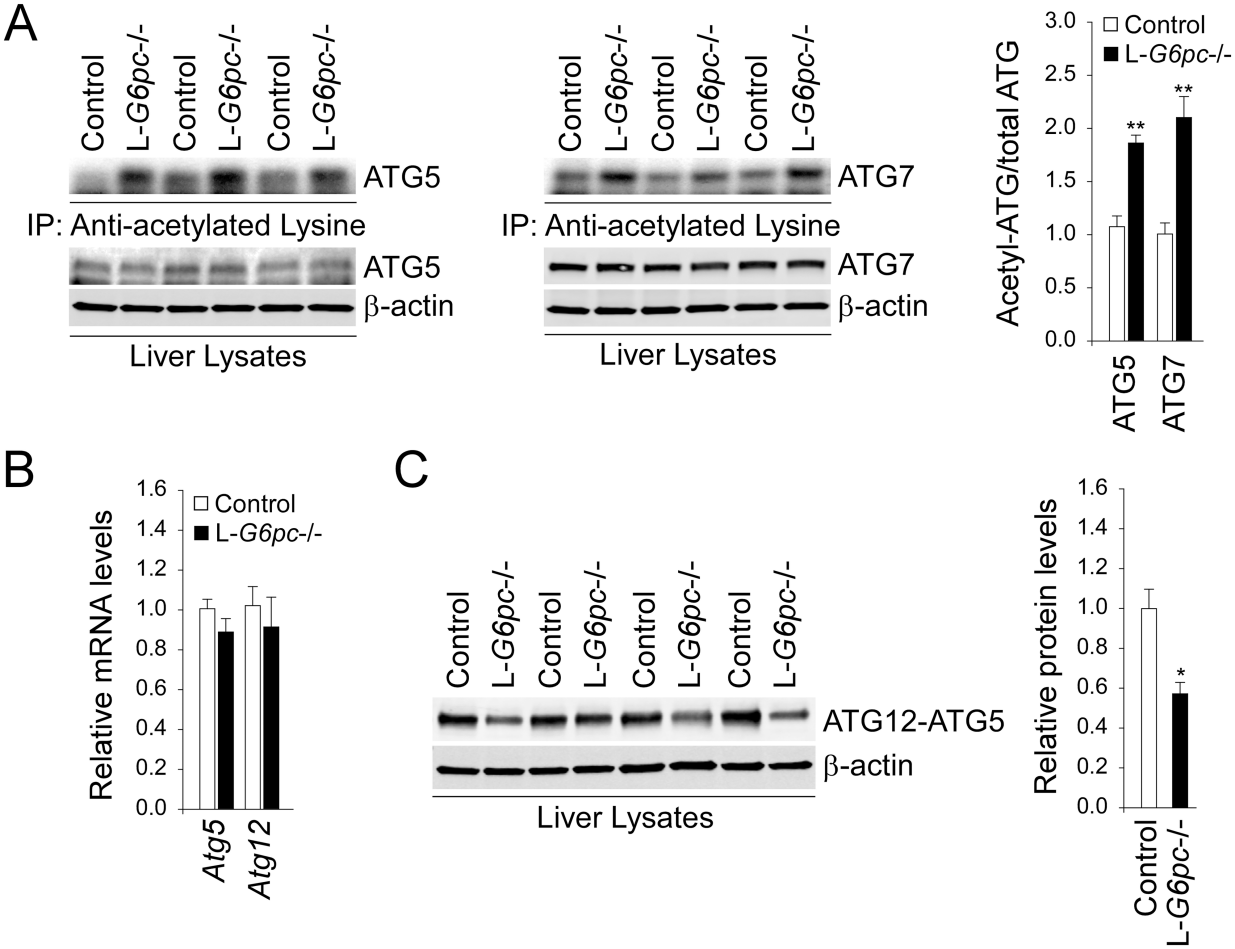
Increased acetylated ATG proteins and decreased ATG5-ATG12 conjugation in the livers of L-*G6pc*-/- mice. (A) Western blots of acetylated and total ATG proteins after immunoprecipitation of liver lysates using anti-acetylated lysine, and quantification of the acetylated ATG proteins/total ATG proteins (n = 4). (B) Quantification of mRNA for hepatic *Atg5* and *Atg12* by real-time RT-PCR (n = 8). (C) Western blots and densitometry analysis of ATG12-ATG5 conjugate and β-actin (n = 4). Data represent the mean ± SEM. *P < 0.05, **P < 0.005.

Studies have shown that acetylation of ATG proteins inhibits the elongation process of autophagosome while SIRT1-mediated deacetylation of ATG proteins positively regulates autophagy [18]. We therefore examined acetylated levels of ATG proteins involved in autophagic vesicle elongation process. During autophagic vesicle elongation, ATG12 is covalently conjugated to ATG5 with the help of ATG7 and ATG10 [8]. The ATG12-ATG5 conjugate interacts with ATG16-like 1 (ATG16L1) and the resulting ATG12-ATG5-ATG16L1 complex promotes another conjugation reaction involved in the conversion from LC3-I to LC3-II, a critical step in autophagosome formation [8]. Consistent with the attenuated expression of SIRT1 deacetylase, the acetylated forms of ATG5 and ATG7 were increased in the livers of L-*G6pc*-/- mice, compared to controls (Fig 3A). While hepatic levels of mRNA for ATG5 and ATG12 were similar between control and L-*G6pc*-/- mice (Fig 3B), hepatic levels of the ATG12-ATG5 conjugate were markedly decreased in the L-*G6pc*-/- mice, compared to controls (Fig 3C), suggesting interference in autophagic vesicle elongation. In summary, the reduced expression of hepatic SIRT1 in L-*G6pc*-/- mice resulted in increased levels of acetylated ATG proteins and decreased levels of the ATG12-ATG5 conjugation that inhibit the elongation of autophagic vesicle.

### Increase in SIRT1 expression corrects hepatic autophagy impairment in L-*G6pc*-/- mice

**Fig 4:**
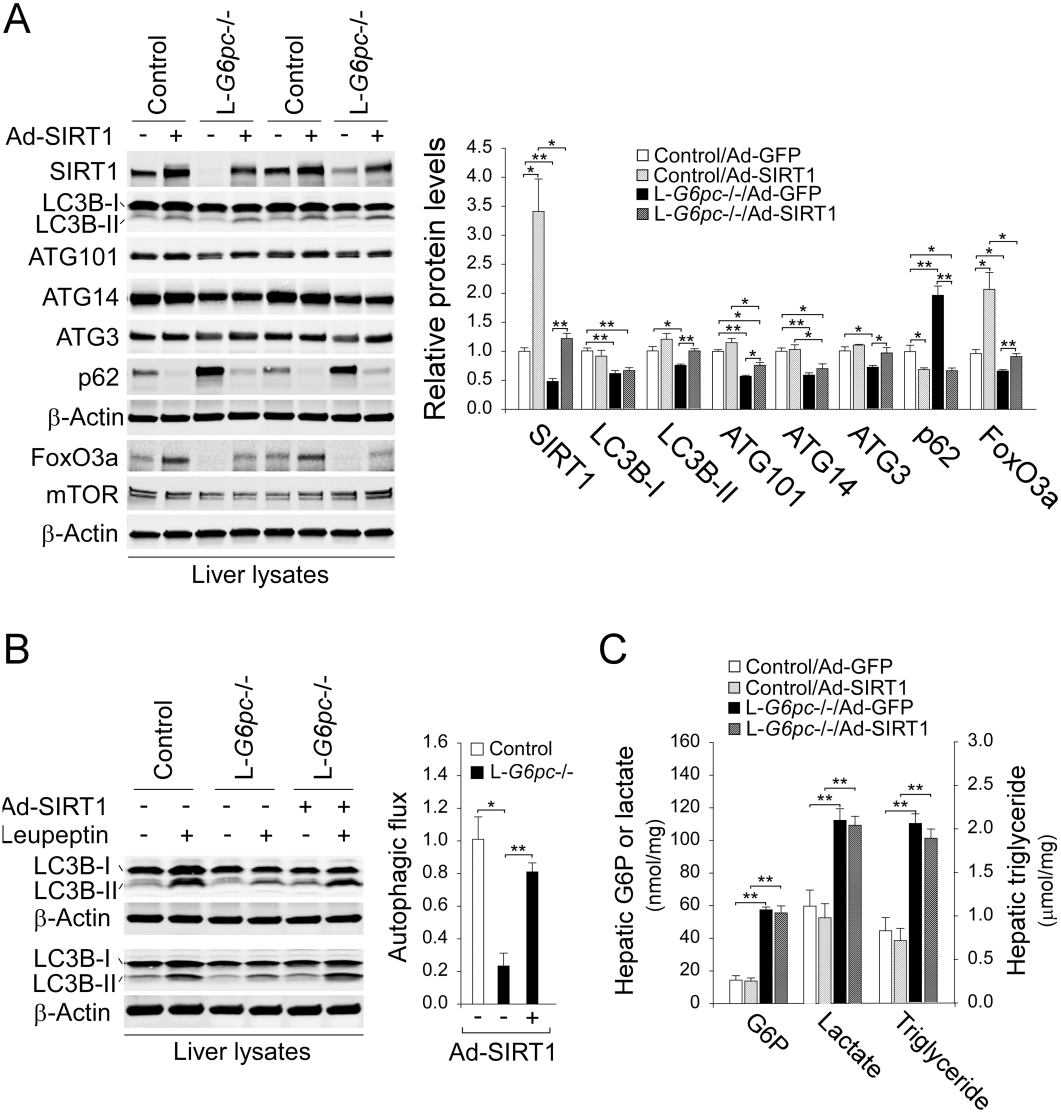
Ad-SIRT1 treatment corrects hepatic autophagy impairment in L-*G6pc*-/- mice. Control and L-*G6pc*-/- mice at 12 WP were treated with 1 x 10^8^ pfu/mice of Ad-GFP or Ad-SIRT1 and analyzed at 13 WP. (A) Western blots for autophagy-related proteins and densitometry analysis (n = 4). (B) Western blots for hepatic LC3B and β-actin in mice that were treated with either saline (-) or leupeptin (+) and determination of autophagic flux (n = 3). (C) The levels of hepatic metabolites in control and L-*G6pc-/-* mice treated with Ad-GFP or Ad-SIRT1 (n = 4). Data represent the mean ± SEM. *P < 0.05, **P < 0.005.

To demonstrate that down-regulation of SIRT1 signaling plays a major role in hepatic autophagy impairment in L-*G6pc*-/- mice, we examined the effect of adenovirus (Ad)-mediated SIRT1 overexpression on autophagy pathway along with Ad-GFP as a control vector. An increase in hepatic SIRT1 expression in L-*G6pc-*/- mice normalized hepatic levels of LC3B-II, ATG101, ATG3, and FoxO3a although the increase in ATG14 was not statistically significant (Fig 4A). Since the autophagy pathway can be regulated by mTOR signaling [7], we also examined the expression of mTOR and our results showed that hepatic levels of mTOR were unchanged in mice overexpressing SIRT1 (Fig 4A). Importantly, hepatic accumulation of p62, indicative of defective autophagy was completely normalized in Ad-SIRT1-treated L-*G6pc*-/- mice (Fig 4A). Furthermore, an increase in SIRT1 expression efficaciously restored the attenuated autophagic flux in the livers of L-*G6pc*-/- mice (Fig 4B). However, SIRT1 overexpression failed to normalize metabolic alterations associated with GSD-Ia including accumulation of hepatic G6P, lactate, and triglyceride in L-*G6pc*-/- mice (Fig 4C). Taken together, down-regulation of SIRT1 signaling underlies the defective hepatic autophagy in L-*G6pc*-/- mice.

### mTOR signaling plays a minimal role in regulating hepatic autophagy in L-*G6pc*-/- mice

**Fig 5:**
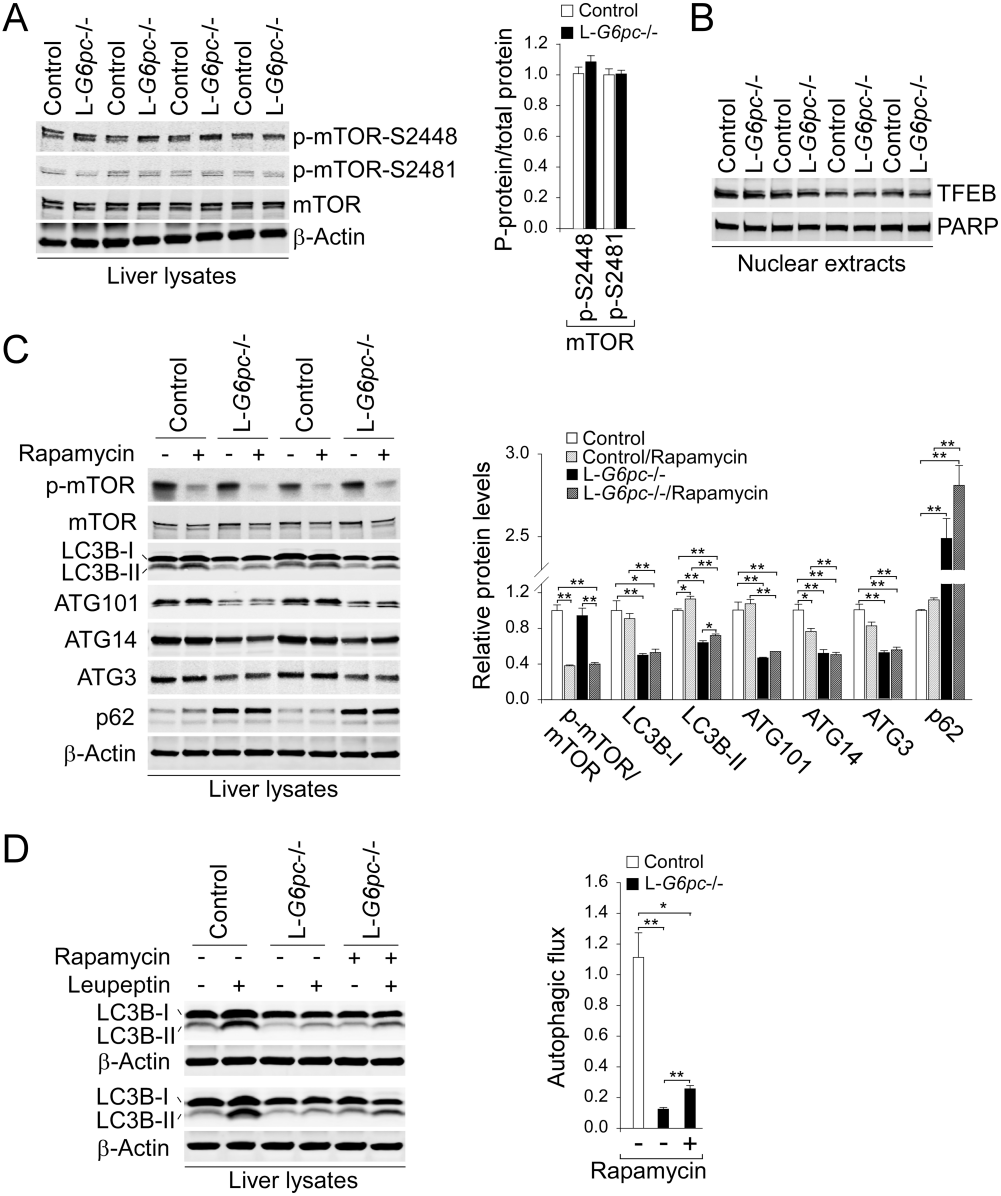
Rapamycin fails to correct impaired hepatic autophagy in L-*G6pc*-/- mice. (A) Western blots for hepatic p-mTOR, mTOR and β-actin, and densitometry analysis (n = 6). (B) Western blot analysis of transcriptional factor EB (TFEB) and Poly (ADP-ribose) polymerase (PARP) in liver nuclear extracts. (C) Western blots and densitometry analysis (n = 4) for autophagy-related proteins in mice treated with either vehicle (-) or 5 mg/kg body weight of rapamycin (+) for 8 consecutive days. (D) Western blots for hepatic LC3B and β-actin in mice treated with rapamycin and leupeptin as indicated, and determination of autophagic flux (n = 3). Data represent the mean ± SEM. *P < 0.05, **P < 0.005.

Farah et al. [11] have recently suggested that activation of mTOR signaling plays a role in autophagy deficiency seen in GSD-Ia. We therefore examined mTOR signaling in the livers of L-*G6pc*-/- mice. Studies have shown that phosphorylated level of mTOR represents its activation status [19–21]. However, hepatic levels of the activated p-mTOR-S2448 and p-mTOR-S2481 were similar between control and L-*G6pc*-/- mice (Fig 5A). mTOR pathway can regulate autophagy via phosphorylation and nuclear exclusion of transcriptional factor EB (TFEB), a transcriptional factor for lysosomal and autophagy gene expression [22, 23]. However, consistent with similar levels of activated mTOR, hepatic levels of nuclear-localized TFEB were also similar between control and L-*G6pc*-/- mice (Fig 5B). Notably, the treatment of rapamycin, a mTOR inhibitor markedly reduced hepatic p-mTOR levels in L-*G6pc*-/- mice but failed to normalize hepatic levels of ATG101, ATG14 and ATG3, although levels of LC3B-II were slightly increased (Fig 5C). The minimal role of mTOR in autophagy deficiency was further supported by similar hepatic levels of p62 between untreated and rapamycin-treated L-*G6pc*-/- mice (Fig 5C). Finally, we showed that the attenuated hepatic autophagic flux in L-*G6pc*-/- mice was only marginally restored by rapamycin (Fig 5D). Taken together, these results support that mTOR plays a minimal role in hepatic autophagy impairment in L-*G6pc*-/- mice.

### rAAV-mediated hepatic G6Pase-α restoration corrects metabolic abnormalities and restores autophagy homeostasis

**Fig 6:**
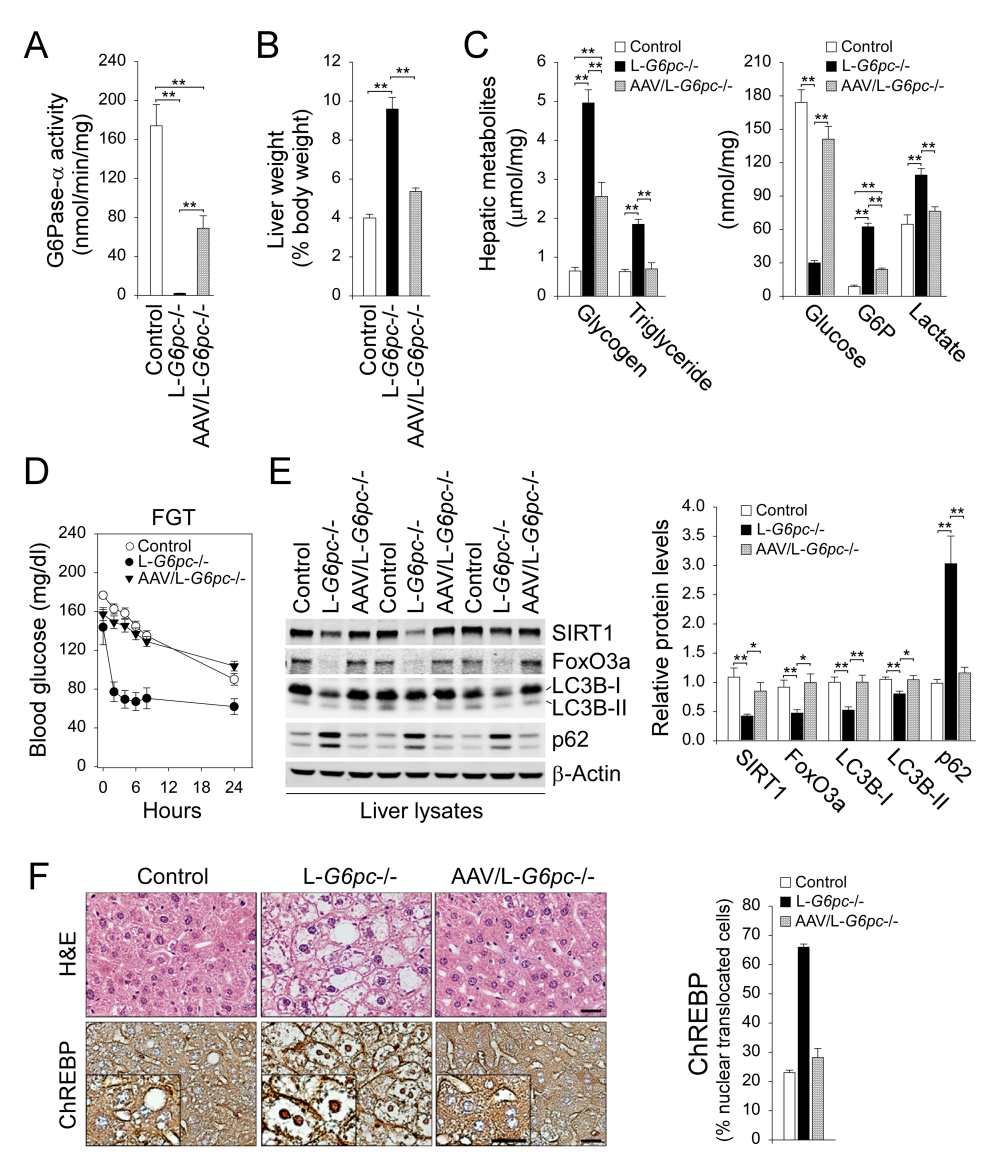
Correction of hepatic G6Pase-α deficiency normalizes autophagy. L-*G6pc*-/- mice were treated with 1 x 10^12^ vp/kg of rAAV-G6PC at 4 WP and analyzed at 12 WP. (A) Hepatic G6Pase-α activity in control (n = 7), L-*G6pc-/-* (n = 7), and rAAV-treated L-*G6pc-/-* (AAV/ L-*G6pc-/-*, n = 8) mice. (B) Liver weights in control (n = 10), L-*G6pc-/-* (n = 5), and AAV/ L-*G6pc-/-* (n = 8) mice. (C) The levels of hepatic metabolites in control, L-*G6pc-/-* and AAV/ L-*G6pc-/-* (n = 8) mice. (D) Fasting glucose test (FGT) profile of control (n = 13), L-*G6pc-/-* (n = 6) and AAV/ L-*G6pc-/-* (n = 8) mice. (E) Western blots of hepatic SIRT1, FoxO3a, LC3B, p62 and β-actin and densitometry analysis (n = 8). (F) Hematoxylin and eosin (H&E) stained liver sections, and immunohistochemical analysis of hepatic ChREBP and quantification of nuclear ChREBP-translocated cells in control, L-*G6pc-/-*, and rAAV-treated L-*G6pc-/-* (AAV/ L-*G6pc-/-*) mice (n = 4). The insets present higher magnification views. Scale bar, 25 μm. Data represent the mean ± SEM. *P < 0.05, **P < 0.005.

SIRT1 overexpression corrected impaired hepatic autophagy but failed to normalize metabolic manifestations associated with GSD-Ia, suggesting that hepatic G6Pase-α plays additional roles in maintaining liver homeostasis. We therefore treated L-*G6pc*-/- mice at 4 weeks post *G6pc* gene deletion (WP) with rAAV-G6PC [24] and examined phenotypic correction of the treated mice at 12 WP. Hepatic G6Pase-α activity in control, L-*G6pc*-/-, and rAAV-treated L-*G6pc*-/- mice averaged 174.1 ± 21.8, 2.0 ± 0.21, and 68.9 ± 12.8 nmol/min/mg, respectively (Fig 6A). We showed that 40% restoration of hepatic G6Pase-α activity was sufficient to normalize liver weights (Fig 6B) as well as hepatic levels of triglyceride, glucose and lactate in the rAAV-treated L-*G6pc*-/- mice, although hepatic levels of glycogen and G6P remained elevated (Fig 6C). Moreover, the rAAV-treated L-*G6pc*-/- mice displayed normal profile of fasting blood glucose (Fig 6D). Importantly, restoration of hepatic G6Pase-α expression completely normalized hepatic levels of SIRT1 and FoxO3a along with normal levels of hepatic LC3B-I, LC3B-II and p62 (Fig 6E). Finally, rAAV-G6PC treatment normalized hepatic levels of nuclear-translocated ChREBP protein and liver histology in the L-*G6pc*-/- mice (Fig 6F). Collectively, hepatic G6Pase-α restoration not only normalizes metabolic abnormalities associated with GSD-Ia but also corrects impaired SIRT1-FoxO signaling and defective autophagy in the livers of L-*G6pc*-/- mice. These results demonstrate that hepatic G6Pase-α plays a critical role in autophagy pathway as well as hepatic metabolisms associated with glucose homeostasis.

## Discussion

**Fig 7:**
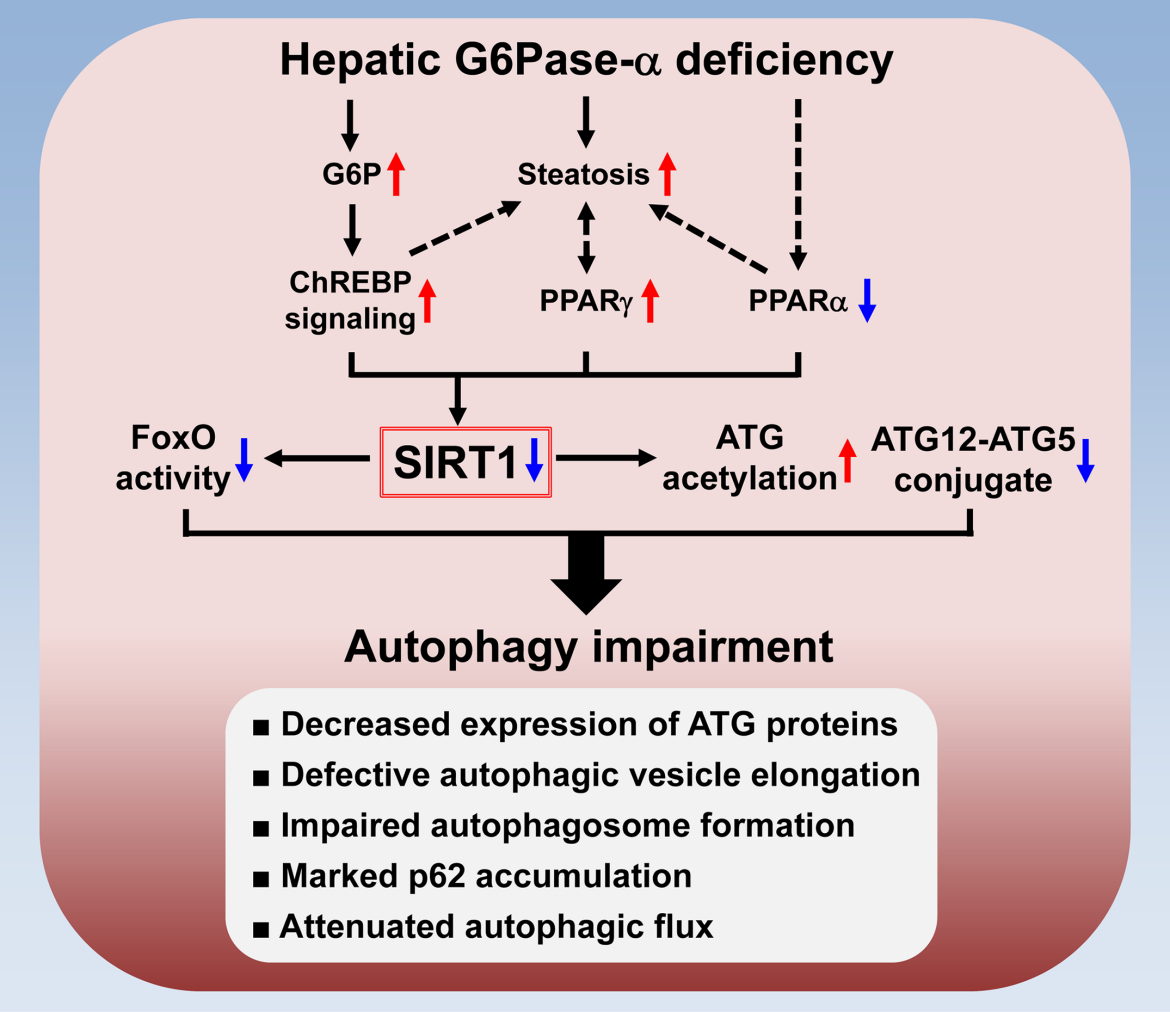
The mechanism underlying autophagy impairment in hepatic G6Pase-α-deficiency. Hepatic G6Pase-α-deficiency leads to metabolic alterations including G6P accumulation and suppressed expression of PPAR-α, a master regulator of fatty acid β-oxidation. The G6P-mediated activation of ChREBP signaling induces lipogenesis, leading to hepatic steatosis which increases the expression of PPAR-γ, another lipogenic factor. Moreover, aberrant PPAR-γ overexpression aggravates hepatic steatosis. The net outcome is downregulation of hepatic SIRT1 signaling. Impaired SIRT1 signaling increases ATG acetylation and decreases ATG12-ATG5 conjugation along with downregulation of FoxO signaling that induces autophagy genes. Accordingly, hepatic G6Pase-α-deficiency-mediated autophagy impairment is characterized by decreased expression of ATG proteins, defective autophagic vesicle elongation, impaired autophagosome formation, marked p62 accumulation and attenuated autophagic flux.

GSD-Ia is a juvenile lethal disease with no curative therapy. Dietary therapies have enabled patients to attain near normal growth and pubertal development but chronic complications remain. Hepatic autophagy deficiency has been linked to many metabolic disorders, including non-alcoholic fatty liver disease and hepatocarcinogenesis. Using adult L-*G6pc*-/- mice, we show that the G6Pase-α-deficient liver displays defective autophagy and reduced expression of SIRT1 and FoxO3a that regulate the expression of many ATG genes. Furthermore, hepatic SIRT1 overexpression corrects defective autophagy in the livers of L-*G6pc*-/- mice, demonstrating that down-regulation of hepatic SIRT1 signaling underlies autophagy deficiency in GSD-Ia (Fig 7). Finally we show that hepatic G6Pase-α restoration normalizes metabolic abnormalities associated with GSD-Ia, restores SIRT1-FoxO signaling, and corrects defective autophagy.

Hepatic autophagy impairment in L-*G6pc*-/- mice is characterized by profound changes in the autophagy system (Fig 7). Firstly, the expression of many ATG proteins involved in autophagy execution, including initiation (ATG101), vesicle nucleation (Beclin-1 and ATG14), elongation (LC3B and ATG3), and mitophagy (BNIP3) was reduced in G6Pase-α-deficient livers. Secondly, autophagic vesicle elongation was defective in the livers of L-*G6pc*-/- mice as evident by increased hepatic levels of acetylated ATG proteins along with reduced levels of the ATG12-ATG5 conjugate. Thirdly, G6Pase-α-deficient livers express reduced levels of LC3B-II along with reduced numbers of autophagic vacuoles, consistent with impaired autophagosome formation. Fourthly, G6Pase-α-deficient livers exhibit marked accumulation of p62, the specific autophagy substrate that plays an important role in tumorigenesis [25]. Consequently, the G6Pase-α-deficient livers display significantly impaired autophagic flux.

It has been reported that levels of cellular lipids negatively regulate the autophagy system [26]. Indeed, the livers of genetically obese (*ob/ob*) and high fat diet-fed (HFD) mice display reduced expressions of many autophagy components [27]. We now show that the primary mechanism underlying impaired hepatic autophagy in L-*G6pc*-/- mice is down-regulation of SIRT1 signaling. SIRT1 activity can be regulated by its altered expression in response to the energy status of the cell as well as by the levels of the cofactor NAD^+^. Indeed, the expression of SIRT1 can be suppressed by lipogenic factors such as ChREBP and PPAR-γ, and stimulated by FoxO1 and PPAR-α in response to nutrient starvation [16]. In L-*G6pc*-/- mice, hepatic NAD^+^ levels were unchanged. On the other hand, liver-specific deletion of G6Pase-α results in suppressed expression of PPAR-α, a master regulator of fatty acid β-oxidation and increased contents of hepatic G6P that activates signaling of ChREBP, a transcriptional activator and repressor [17]. Activation of ChREBP is associated with increased lipogenesis, leading to a marked increase in hepatic steatosis that is known to increase the expression of PPAR-γ [28], a lipogenic factor capable of suppressing SIRT1 expression [16]. Moreover, aberrant PPAR-γ overexpression has been shown to aggravate hepatic steatosis [29, 30]. The net outcome was a decrease in the expression of SIRT1 in the livers of L-*G6pc*-/- mice, providing a possible link between hepatic steatosis and defective autophagy in GSD-Ia (Fig 7). Studies have shown that PPAR-α positively regulates hepatic autophagy [31, 32], suggesting that downregulation of PPAR-α in G6Pase-α-deficient liver also contributes to defective autophagy.

Autophagy can also be regulated by mTOR signaling [7]. Recently, Farah et al. [11] showed that inhibition of mTOR signaling by rapamycin increased the expression of LC3-II and autophagic vesicles in the livers of young global *G6pc*-/- mice and suggested that activation of mTOR signaling may underlie hepatic autophagy deficiency in GSD-Ia. However, several lines of evidence showed that mTOR pathway plays a minimal role in hepatic autophagy impairment in L-*G6pc*-/- mice. Firstly, hepatic levels of the activated p-mTOR-S2448 and p-mTOR-S2481 as well as hepatic levels of nuclear TFEB, a target of mTOR signaling were similar between control and L-*G6pc*-/- mice. Secondly, the livers of rapamycin-treated L-*G6pc*-/- mice continued to express reduced levels of LC3B-I, ATG101, ATG14, ATG3 and marked high levels of p62 similar to untreated L-*G6pc*-/- mice. Thirdly, rapamycin treatment marginally restored hepatic autophagic flux in L-*G6pc*-/- mice. The major difference between two studies is the age of the animals used: adult L-*G6pc*-/- mice were used in this study and 10-day-old *G6pc*-/- mice were used by Farah et al [11]. It has been reported that depending on developmental stages, hepatic gene expression is differentially regulated by mTOR [33] and that milk intake can activate mTOR signaling during postnatal lactation period [34]. Therefore, it is possible that mTOR signaling is activated in young *G6pc*-/- mice during early postnatal development. However, the inability of rapamycin to normalize the expression of ATG proteins and p62 strongly supports the lesser role of mTOR signaling in autophagy deficiency in adult L-*G6pc*-/- mice.

While young *G6pc*-/- mice exhibited early signs of hepatic autophagy impairment [11], adult L-*G6pc*-/- mice displayed many aspects of hepatic autophagy impairment along with a marked increase of p62, a selective autophagy substrate that accumulates in premalignant livers and hepatic tumors [35]. Studies have shown that autophagy-deficient mice develop HCA and the sustained p62 accumulation contributes to the development of HCA/HCC [12, 13], the hallmark of long-term complication of GSD-Ia. Thus, the L-*G6pc*-/- mouse that manifests hepatic autophagy deficiency and develops HCA is an excellent model to study the etiology and therapies of HCA in GSD-Ia.

Our study establishes an important role of SIRT1 in maintaining autophagy function in G6Pase-α-deficient liver (Fig 7). The transcription of SIRT1 can be stimulated by PPAR-α, and down-regulated by PPAR-γ and ChREBP [16]. Therefore, modulation of these factors in the liver may improve hepatic autophagy impairment in the L-*G6pc*-/- mice. Notably, the G6P-mediated activation of hepatic ChREBP signaling can be reversed by rAAV-G6PC-mediated gene therapy that restores hepatic G6Pase-α expression. On the other hand, hepatic autophagy can be stimulated by a PPAR-α agonist [31]. Therefore, pharmacological interventions using PPAR-α agonists offers another avenue to improve hepatic autophagy impairment in the L-*G6pc*-/- mice.

We have shown that systemic administration of rAAV-G6PC to young global *G6pc-/-* mice delivers the G6Pase-α transgene to the liver and corrects metabolic abnormalities [24, 36]. When followed out to 70–90 week-old, the rAAV-G6PC-treated *G6pc-/-* mice maintain glucose homeostasis and show no evidence of HCA/HCC [36]. We now show that rAAV-mediated G6Pase-α restoration in adult L-*G6pc*-/- mice corrects metabolic abnormalities associated with GSD-Ia and completely normalizes hepatic autophagy deficiency that contributes to HCA development. Taken together, our results suggest that gene therapy offers a promising therapeutic strategy to rectify impaired autophagy and to prevent HCA development in GSD-Ia.

## Materials and methods

### Ethics statement

All animal studies were conducted under an animal protocol (ASP-16-086) approved by the Eunice Kennedy Shriver National Institute of Child Health and Human Development Animal Care and Use Committee followed the guidelines (https://oacu.oir.nih.gov/animal-research-advisory-committee-guidelines).

### Animals

The *G6pc* ^*fx/fx*^ mice containing exon 3 of the *G6pc* gene flanked with *loxP* sites [37] were crossed with the SA^creERT2/w^ mice expressing a tamoxifen-dependent Cre-recombinase under the control of the serum albumin promoter [38]. The liver-specific *G6pc*-deficient (L-*G6pc*-/-) and L-*G6pc*+/- mice were generated by tamoxifen-mediated excision of the *G6pc* exon 3 in 6-week-old *G6pc*^fx/fx^.SA^creERT2/w^ and *G6pc*^fx/w^.SA^creERT2/w^ mice, respectively, as previously described [3]. GSD-Ia is an autosomal recessive disorder and the phenotypes of L-*G6pc*+/+ and L-*G6pc*+/- were indistinguishable, therefore both mice were used as controls. To reconstitute hepatic G6Pase-α activity, rAAV-G6PC, a rAAV vector expressing human G6Pase-α [24] at 1 x 10^12^ viral particles/kg was infused into L-*G6pc*-/- mice via retro-orbital sinus at 4 WP (weeks post *G6pc* gene deletion). Liver samples were collected from mice at 12WP following a 6-hour fast.

### Adenovirus vector and rapamycin treatment

The recombinant adenovirus vectors expressing human SIRT1 (Ad-SIRT1) and GFP (Ad-GFP) obtained from Vigene Biosciences (Rockville, MD) were amplified using 293 cells and purified via CsCl gradient centrifugation, The CsCl-purified vectors were then dialyzed against a buffer containing 10 mM Tris-HCL, pH 7.4, 1 mM MgCl_2_, and 10% glycerol. Control and L-*G6pc*-/- mice at 12 WP were infused with either Ad-GFP or Ad-SIRT1 via retro-orbital sinus at 1 x 10^8^ pfu/mice and their phenotype was analyzed at 13 WP. Ad-GFP was used as a control vector. For rapamycin treatment, control and L-*G6pc*-/- mice at 12 WP were injected intraperitoneally with rapamycin (LC Laboratories) at 5 mg/kg body weight in vehicle (1% polyethylene glycol, and 1% Tween-80, Sigma-Aldrich) for 8 consecutive days. As controls, the mice were injected with vehicle alone.

### Autophagy flux determination

Autophagic flux determination was performed as previously reported [39]. Briefly, control and L-*G6pc*-/- mice were fasted for 20 hours to induce autophagy pathway. Then, the mice were injected intraperitoneally with saline or leupeptin (Sigma-Aldrich, 40 mg/kg body weight) that blocks lysosomal degradation, and were sacrificed 4 hours later. LC3B-II and β-actin in liver lysates were analyzed by Western blots and quantified by densitometry. The protein levels of LC3B-II were normalized against β-actin. Autophagic flux was determined by the difference in normalized LC3B-II protein levels between in mice treated with saline and in mice treated with leupeptin.

### Metabolites determinations

Liver lysates were deproteinized using 14% (wt/vol) perchloric acid, and then neutralized with 2 M KOH/0.2 M MOPS. The levels of glucose, G6P, and lactate in deproteinized lysates were determined using the respective assay kit from BioVision (Mountain View, CA). Hepatic levels of NAD^+^ and triglyceride were determined using the EnzyChrom NAD^+^/NADH assay kit (BioAssay Systems, Hayward, CA) and a Triglyceride Quantification Kit (Biovision), respectively. Hepatic glycogen levels, microsome isolation, and G6Pase-α activity assay were performed as described [24].

### Primary hepatocyte isolation

Hepatocytes were isolated from control and L-*G6pc*-/- mice at 12 WP using a two-step collagenase perfusion method. Liver was perfused via the portal vein with liver perfusion medium (Gibco, Waltham, MA) for 5 min at 37°C, followed by liver digest medium (Gibco) for 5 min at 37°C. The excised liver was incubated in liver digest medium for 30 min at 37°C, and then passed through a 100 μm cell strainer (Falcon, Franklin Lakes, NJ). The hepatocytes were pelleted by centrifugation at 4°C, washed twice with hepatocyte wash medium (Gibco), and purified via 20% Percoll gradients (GE Healthcare, Waukesha, WI). The resulting hepatocytes were washed with Willams E medium (Gibco) and resuspended in HepatoZYME-SFM (Gibco).

### Flow cytometry analysis

To determine autophagy vacuoles, 2 X 10^5^ hepatocytes were incubated with 1 μl Cyto-ID Green Autophagy detection reagent (Enzo Life Sciences, Exeter, United Kingdom) in 1 ml of HepatoZYME-SFM for 30 min at 37°C, washed, and analyzed by flow cytometry using a Guava EasyCyte Mini System (Millipore, St Charles, MO).

### Quantitative real-time RT-PCR and western blot analyses

The expression of mRNA was quantified by real-time PCR using the TaqMan probes (Life Technologies) in an Applied Biosystems 7300 Real-Time PCR system. Data were analyzed with the SDS Version1.3 software (Applied Biosystems) and normalized to Rpl19 RNA. Western blot images were detected with the use of the LI-COR Odyssey scanner and the Image studio 3.1 software (Li-Cor Biosciences, Lincoln, NE). The antibodies were purchased from Cell Signaling Technology: PARP (#9542), Acetylated-Lysine (#9814), FoxO3a (#12829), Beclin-1 (#3738), ATG3 (#3415), ATG101 (#13492), BNIP3 (#12396), ATG7 (#8558), mTOR (#2983), p-mTOR-S2448 (#5536), and p-mTOR-S2481 (#2974). The antibodies were purchased from Abcam: LC3B (ab51520), p62 (ab91526), ATG14 (ab139727), and TFEB (ab122910). The antibodies were purchased from Santa Cruz Biotechnology: ATG5 (sc-515347), β-actin (sc-47778), PPAR-γ (sc-7196), and PPAR-α (sc-9000). SIRT1 (#07–131) antibody was purchased from Millipore. The monoclonal antibody against human G6Pase-α was raised in mice using a peptide containing amino acid residues 227 to 268 in luminal loop 3 of human G6Pase-α [40]. Antigen injection, hybridoma generation, and clone screening were performed by A&G Pharmaceutical, Inc. Hybridoma clones were screened using the enzyme-linked immunosorbent assay (ELISA) on the immunogen. The culture supernatant from a hybridoma clone (3A9) showing high sensitivity to the immunogen was subjected to affinity purification using the peptide coupled agarose. The specificity of the purified antibody was confirmed by ELISA.

### Immunoprecipitation

Liver tissues were homogenized with the IP lysis buffer (25 mM Tris-HCl, pH 7.4, 150mM NaCl, 1% NP-40, 1 mM EDTA and 5% glycerol) containing 1 X Halt Protease and Phosphatase Inhibitor Cocktails (Thermo Scientific) and centrifuged at 12000 g for 20 min at 4°C. The resulting supernatants were subjected to immunoprecipitation with the indicated antibody. To detect acetylated FoxO3a, liver nuclear extracts prepared using the NE-PER Nuclear and Cytoplasmic Extraction Kit (Thermo Scientific, Waltham, MA) were subjected to immunoprecipitation with an antibody against FoxO3a (Cell Signaling) and the resulting precipitates were examined by Western blot analysis with the antibody against acetylated lysine (Cell Signaling).

### Electron microscopy

Mouse livers were fixed in 0.1 M sodium cacodylate buffer (pH 7.4) containing 2.5% glutaraldehyde for 1 h at room temperature. The fixed liver tissues were then treated with 1% osmium tetroxide in 0.1M sodium cacodylate buffer and 2% uranyl acetate for 1 h at room temperature. The liver tissues were then serially dehydrated by ethanol, and then serially infiltrated via Spurr’s resin/ethanol up to 100% resin which was then polymerized for 18 h at 70°C in a Pelco BioWave Pro microwave oven (Ted Pella, Inc., Redding, CA), and finally cut into 90 nm sections using a Reichert-Jung Ultracut-E ultramicrotome. The resulting grids were stained with uranyl acetate and lead citrate, and imaged with a JEOL-1400 transmission electron microscope operated at 80 kV.

### Immunohistochemical analysis

Mouse liver tissues were fixed in 10% neutral buffered formalin (Fisher Scientific, Grand Island, NY), embedded in paraffin, then sectioned to 10 μm thickness, and the paraffin was removed by Xylene (Fisher Scientific). Liver sections were then incubated in antigen unmasking solution (Vector Laboratories, Burlingame, CA) for 10 min at 100°C. Endogenous peroxidases were quenched with 0.9% hydrogen peroxide in methanol, and then blocked with the Avidin/Biotin Blocking Kit (Vector Laboratories). The sections were then incubated with the anti-ChREBP (NOVUS) antibody and followed with the biotinylated secondary antibodies (Vector Laboratories). The resulting complexes were detected with an ABC kit using the DAB Substrate (Vector Laboratories). Sections were also counterstained with hematoxylin (Sigma-Aldrich) and visualized using a Zeiss Axioskop2 plus microscope equipped with 10X/0.45NA, 20X/0.5NA or 40X/0.75NA objectives (Carl Zeiss, Oberkochen, Germany). Nuclear translocalization of ChREBP was quantified by calculating the percentages of hepatocytes containing ChREBP-positive nuclei in 10 randomly selected fields of the livers stained with ChREBP antibody at 400 x magnification.

### Statistical analysis

The unpaired *t* test was performed by using the GraphPad Prism Program, version 4 (San Diego, CA). The values were considered statistically significant at *P* < 0.05.

## Supporting information

S1 Fig Characterization of L-*G6pc*-/- mice. (A) PCR analysis of genomic DNA. The 320-bp band denotes Cre recombinase, the 103 bp band denotes wild-type allele, and the 162 bp band denotes the floxed allele. (B) Western blots of G6Pase-α and β-actin in the liver, kidney and muscle of age-matched control and L-*G6pc-/-* mice.
(TIF)

S2 Fig Hepatic levels of nuclear and cytoplasmic ChREBP. Western blots of nuclear and cytoplasmic ChREBP in the livers of control and L-*G6pc-/-* mice and densitometry analysis (n = 4). Data represent the mean ± SEM. *P < 0.05, **P < 0.005.
(TIF)
