# Supplementary figures and images for: Downregulation of SIRT1 signaling underlies hepatic autophagy impairment in glycogen storage disease type Ia
Source: PLoS Genet. 2017 May 30;13(5):e1006819. doi: 10.1371/journal.pgen.1006819 (PMC5469511; doi:10.1371/journal.pgen.1006819)

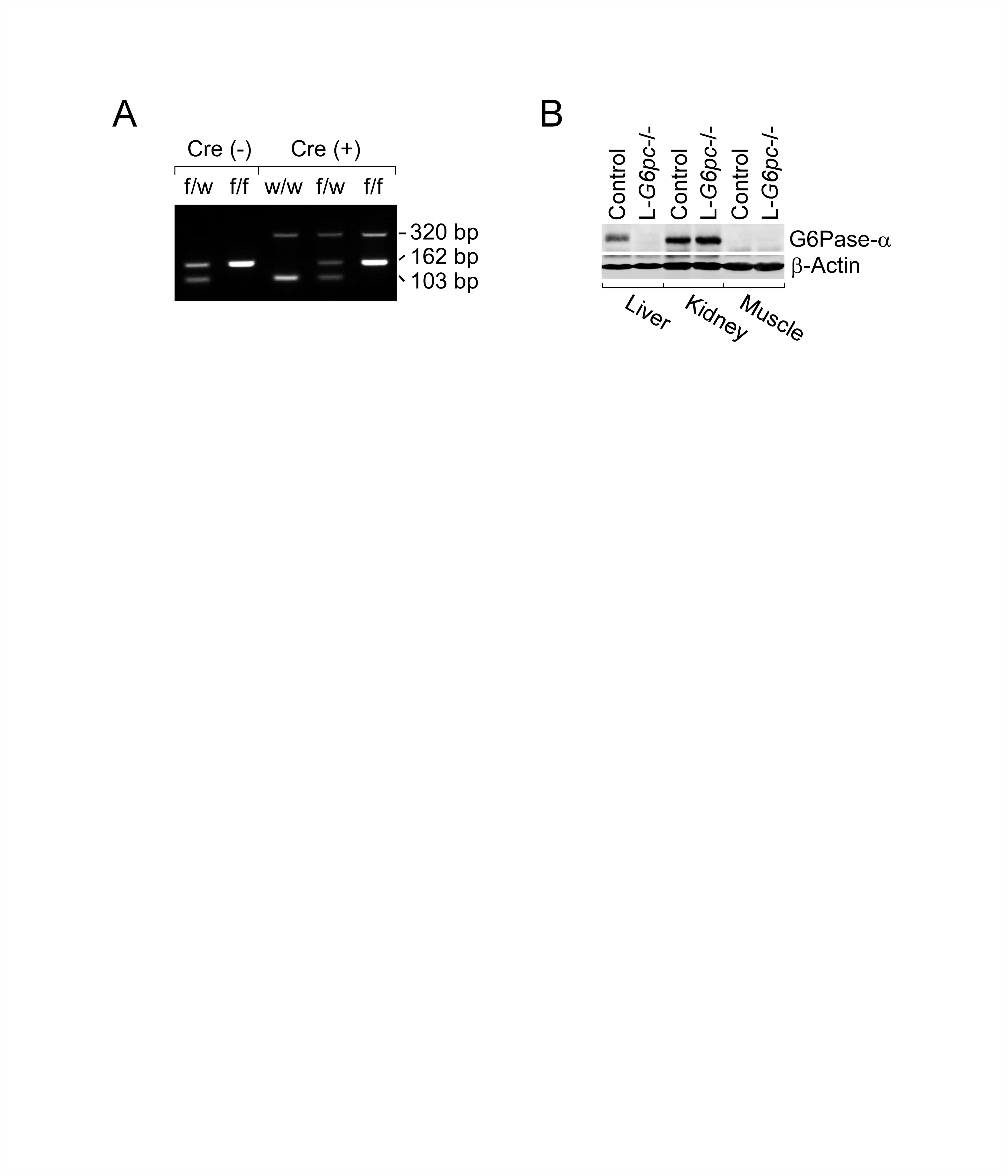

Supplement: S1 Fig — (A) PCR analysis of genomic DNA. The 320-bp band denotes Cre recombinase, the 103 bp band denotes wild-type allele, and the 162 bp band denotes the floxed allele. (B) Western blots of G6Pase-α and β-actin in the liver, kidney and muscle of age-matched control and L-G6pc-/- mice. (TIF) [file pgen.1006819.s001.tif]

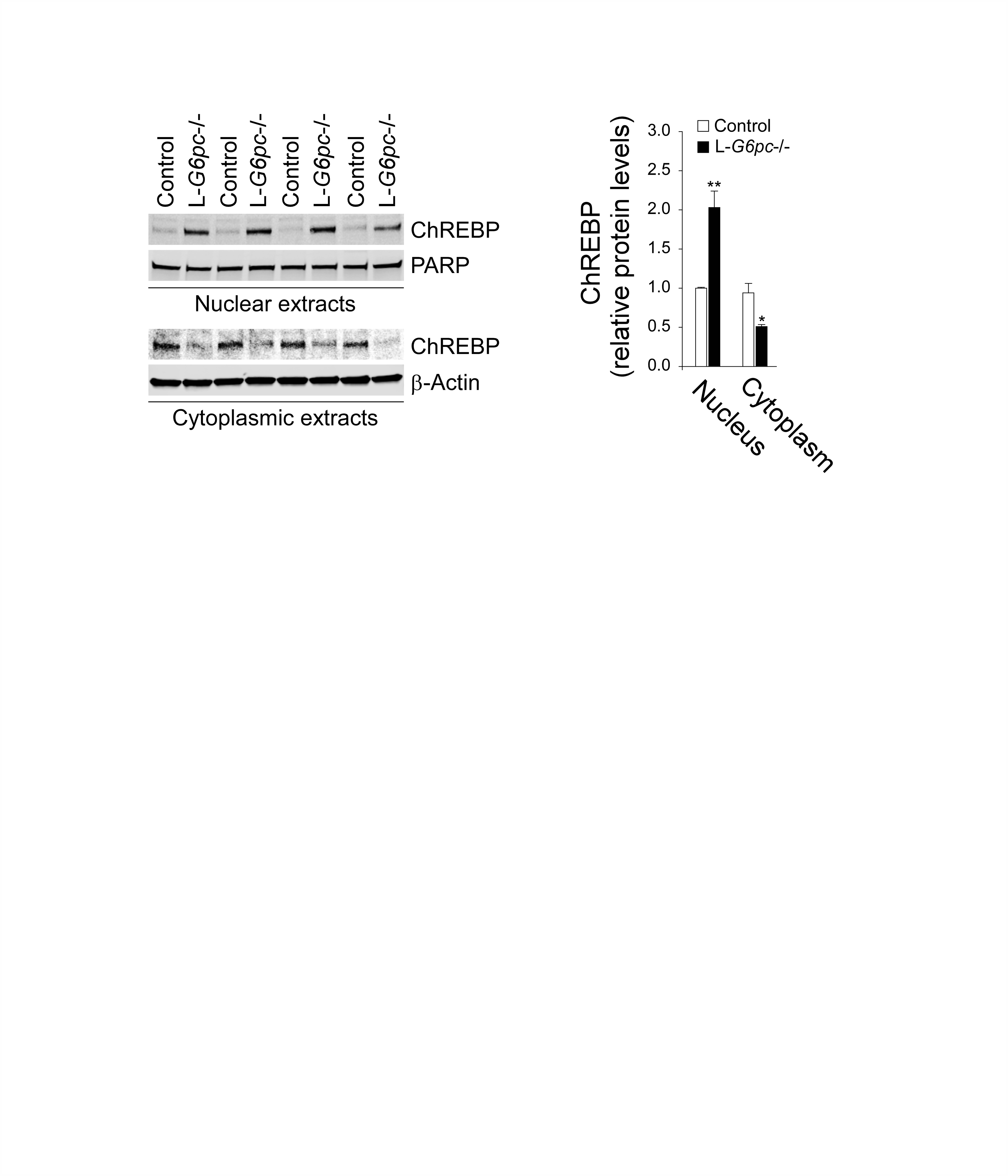

Supplement: S2 Fig — Western blots of nuclear and cytoplasmic ChREBP in the livers of control and L-G6pc-/- mice and densitometry analysis (n = 4). Data represent the mean ± SEM. *P < 0.05, **P < 0.005. (TIF) [file pgen.1006819.s002.tif]
